# Distinct stem cell identities converge into shared erythroid stress in ERCC6L2 disease and Shwachman–Diamond syndrome

**DOI:** 10.1002/hem3.70374

**Published:** 2026-06-16

**Authors:** Laura Langohr, Ilse Kaaja, Suvi P. M. Douglas, Hanna Nebelung, Jessica Koski, Ina Ikonen, Lotta Katainen, Katri Maljanen, Marja Hakkarainen, Tuulia Räisänen, Riitta Niinimäki, Sakari Kakko, Timo Siitonen, Sadiksha Adhikari, Markus Vähä‐Koskela, Caroline A. Heckman, Jenni Lahtela, Ulla Wartiovaara‐Kautto, Esa Pitkänen, Outi Kilpivaara

**Affiliations:** ^1^ Applied Tumor Genomics Research Program, Research Programs Unit, Faculty of Medicine University of Helsinki Helsinki Finland; ^2^ Institute for Molecular Medicine Finland (FIMM), HiLIFE University of Helsinki Helsinki Finland; ^3^ Department of Medical and Clinical Genetics, Medicum University of Helsinki Helsinki Finland; ^4^ iCAN Digital Precision Cancer Medicine Flagship Helsinki Finland; ^5^ Department of Hematology, Helsinki University Hospital Comprehensive Cancer Center University of Helsinki Helsinki Finland; ^6^ Department of Pediatrics, Oulu University Hospital and PEDEGO Research Unit University of Oulu Oulu Finland; ^7^ Cancer Center, Oulu University Hospital Oulu Finland; ^8^ Research Unit of Biomedicine and Internal Medicine University of Oulu Oulu Finland; ^9^ HUSLAB Laboratory of Genetics HUS Diagnostic Center (Helsinki University Hospital) Helsinki Finland; ^10^ K. Albin Johansson Cancer Research Fellow, Foundation for the Finnish Cancer Institute

## Abstract

ERCC6L2 disease (ED) is a rare bone marrow failure syndrome caused by biallelic germline mutations in *ERCC6L2*. ED leads to the accumulation of somatic *TP53* mutations, myelodysplastic syndrome, and acute myeloid leukemia (AML) with erythroid predominance and poor prognosis. While ERCC6L2 is implicated in DNA replication and repair, the transcriptomic events underlying delayed erythropoiesis and leukemic progression remain largely undefined. To delineate these processes, we leverage bulk and single‐cell transcriptomics of patient fibroblasts, bone marrow, and peripheral blood across disease stages, including single‐cell *TP53* genotyping. We identify disease‐associated erythroid dysregulation and ferroptotic stress emerging prior to *TP53* mutation, highlighting an early vulnerability in ED leukemogenesis. We compare ED to Shwachman–Diamond syndrome (SDS) to reveal shared and disease‐specific transcriptional programs. *TP53* mutations in ED and SDS arise in hematopoietic stem and progenitor cells but do not independently drive changes in cell cycle or stress pathways during erythropoiesis, despite harboring distinct germline defects. Both diseases converge in late erythropoiesis into a stress state characterized by ferroptotic signaling, G1 arrest, and *BCL2L1* upregulation. As a disease‐specific pattern, ED shows aberrant erythroid priming with *TP53*‐driven differentiation arrest shaping progression toward erythroid leukemia. Thereby, we establish the first patient‐level single‐cell map of ED and provide a curated resource for future work on ED, SDS, and *TP53*‐driven leukemogenesis. Overall, our findings in pre‐malignant ED offer a window into early alterations leading to high‐risk leukemia.

## Graphical abstract

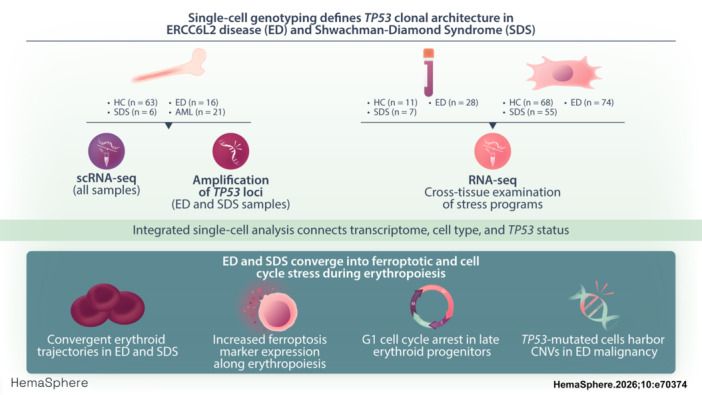

## INTRODUCTION

ERCC6L2 disease (ED) is an inherited bone marrow failure (BMF) syndrome caused by biallelic germline mutations in *ERCC6L2*.^1^, ^2^, ^3^ ED patients are predisposed to primarily erythroid, lineage‐restricted myeloid malignancy.^4^, ^5^ ERCC6L2 functions in DNA repair,^1^, ^2^, ^6^, ^7^, ^8^ centromere stability,^9^ mitochondrial homeostasis,^1^ regulation and facilitation of transcription,^8^ and class‐switch recombination.^10^ Clinically, ED often presents with BMF characterized by mild to moderate peripheral blood (PB) cytopenias.^5^ As the condition progresses, multiple *TP53*‐mutated hematopoietic clones develop as somatic compensation to declining bone marrow (BM) function.^11^, ^12^ This evokes a high risk of loss of heterozygosity, and biallelic *TP53* inactivation, a pivotal event that propels the development of an aggressive, erythroid‐predominant myeloid malignancy. Despite significant efforts to find cures for *TP53*‐mutated myeloid malignancies, their prognosis is dismal.^13^ Particularly for ED‐related hematological malignancy if diagnosed at the final, leukemic stage, the outcomes are universally unfavorable.^5^

In light of these challenges, our study shifts focus from malignancy to earlier disease stages by investigating the transcriptomic landscape of *ERCC6L2*‐deficient hematopoiesis, particularly during BMF. By identifying biological aberrations caused by *ERCC6L2* germline defects, we uncover insights for future studies on potential therapeutic targets. *ERCC6L2* deficiency in hematopoietic stem and progenitor cells (HSPCs) leads to delayed erythroid differentiation.^14^ However, how somatic *TP53* alterations influence lineage‐specific transcriptional programs and differentiation trajectories during BMF in ED remains incompletely understood. Recent functional studies in murine cells demonstrated that Ercc6l2 loss induces replication stress and DNA damage in HSPCs, leading to impaired erythroid differentiation and BMF. Trp53 inactivation restored hematopoiesis, but permits leukemic transformation.^15^ Our goal is to identify disease‐specific transcriptional programs in ED by comparing it to Shwachman–Diamond syndrome (SDS), another inherited BMF syndrome with a propensity for *TP53*‐mutated myeloid malignancies.^11^, ^16^ Leveraging an exceptionally comprehensive dataset for a rare disease, we analyzed erythropoiesis in ED patients by comparing their PB and BM cell transcriptomes to those of SDS patients and healthy controls. Within the BM, we further compared *TP53*‐mutated and wild‐type cells. We complemented these analyses with transcriptomic profiling of patient‐derived fibroblasts to uncover germline‐driven effects of *ERCC6L2* and *SBDS* deficiency, independent of somatic *TP53* alterations.

## MATERIALS AND METHODS

Patient information, including *TP53* mutation status, was collected from the Finnish Hematological Registry and Biobank, and clinical repositories. The study was conducted in accordance with the Declaration of Helsinki. The study has been approved by Helsinki University Central Hospital ethics review committee (#206/13/03/03/2016, amendment 2023, and HRUHLAB2). All samples from living individuals are derived after written informed consent.

**Figure 1:**
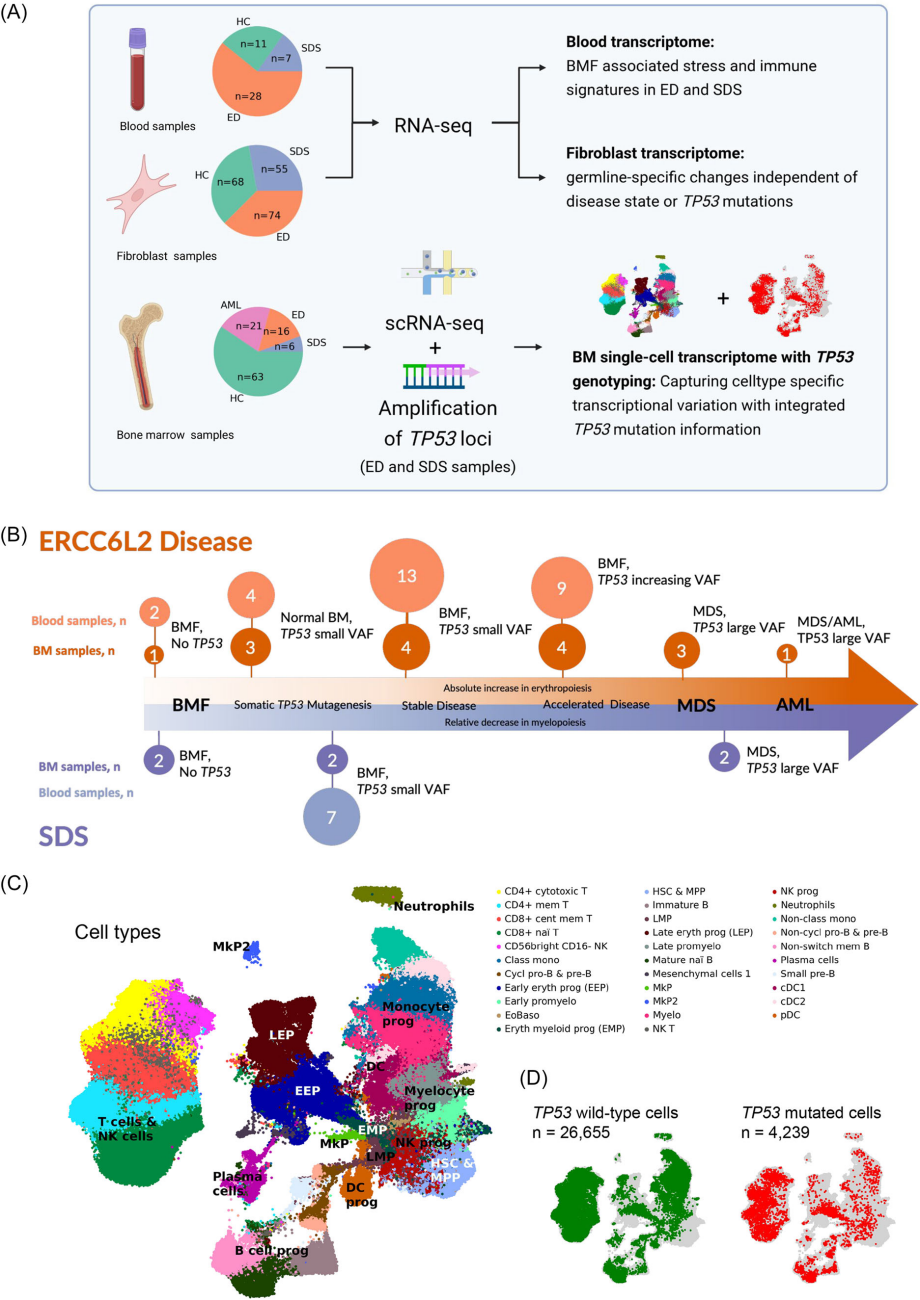
**Data summary. (A)** Schematic of sample and data processing. Numbers denote the number of samples; amplification of *TP53* loci enabled targeted genotyping of known *TP53* mutation sites (Supporting Information S2: Methods, Supplemental Data). Image created with BioRender. **(B)** ERCC6L2 disease (ED) and Shwachman–Diamond (SDS) blood and bone marrow (BM) samples included in this study, depicted at their different stages of disease. Numbers denote the number of samples obtained from 10 ED and 5 SDS patients for BM, and from 12 ED and 5 SDS patients for blood. Colors depict the type of samples (BM or blood). Samples with “no *TP53*” denote samples without somatic *TP53* mutations, and other samples depict cases with 1–4 *TP53* mutations. **(C)** Detected cell types of our integrated single‐cell transcriptomics data. **(D)** *TP53* mutation status of cells. Numbers denote the number of cells that were identified as *TP53*‐mutated or *TP53* wild‐type. AML, acute myeloid leukemia; BMF, bone marrow failure; HC, healthy control; HSC, hematopoietic stem cell; LMP, lymphomyeloid progenitor; MDS, myelodysplastic syndrome; MPP, multipotent progenitor cell; VAF, variant allele fraction.

Patient samples, mostly treatment‐naïve diagnostic specimens, were collected in conjunction with diagnostic samples and are depicted in Figure 1A,B, and detailed in Supporting Information S3: Tables 1 and 2. We examined the transcriptome of 16 BM samples from 10 patients with ED, all carrying the homozygous Finnish founder mutation in *ERCC6L2* (NM_020207.7: c.1424delT, p.Ile475ThrfsTer36, rs768081343).^3^, ^4^, ^5^, ^14^ Samples were stratified according to clinical diagnosis at the time of sampling into pre‐myelodysplastic syndrome (MDS; BMF or morphologically normal BM), MDS, and MDS/acute myeloid leukemia (AML) disease stages. For comparison, we included 6 BM samples from 5 patients with SDS, 20 BM samples from 20 AML patients without erythroid predominance (Supporting Information S3: Table 3), and 63 BM samples from 8 Human Cell Atlas (HCA) control donors^17^ (8 samples per donor, except for one only 7) and 1 erythroleukemia (AML M6) case.^18^

Somatic mutation profiling, including identification of *TP53* mutations and their variant allele fractions (VAFs), was performed as part of clinical diagnostics using a targeted myeloid mutation sequencing panel (Supporting Information S2: Supplemental Methods). ED and SDS patients carried up to four *TP53* mutations in their BM (Supporting Information S3: Tables 1 and 2), most of them located in hotspots (Supporting Information S1: Figure 1). Chromosomal aberrations were identified as part of routine clinical diagnostic work‐up using conventional cytogenetic and/or molecular techniques at the patients' centers of care.

We further examined the blood transcriptome of 28 samples from 12 ED patients, 7 samples from 5 SDS patients, and 11 samples from 9 controls; and the skin fibroblast transcriptome of 74 samples from 8 ED patients, including 1 patient with a compound heterozygous mutation (the above‐mentioned founder mutation and *ERCC6L2* NM_020207.7: c.4454_4455delGA, p.Arg1474ThrfsTer10, rs1553528966), 55 samples from 4 SDS patients, and 68 samples from 8 controls (Figure 1A,B).

**Figure 2:**
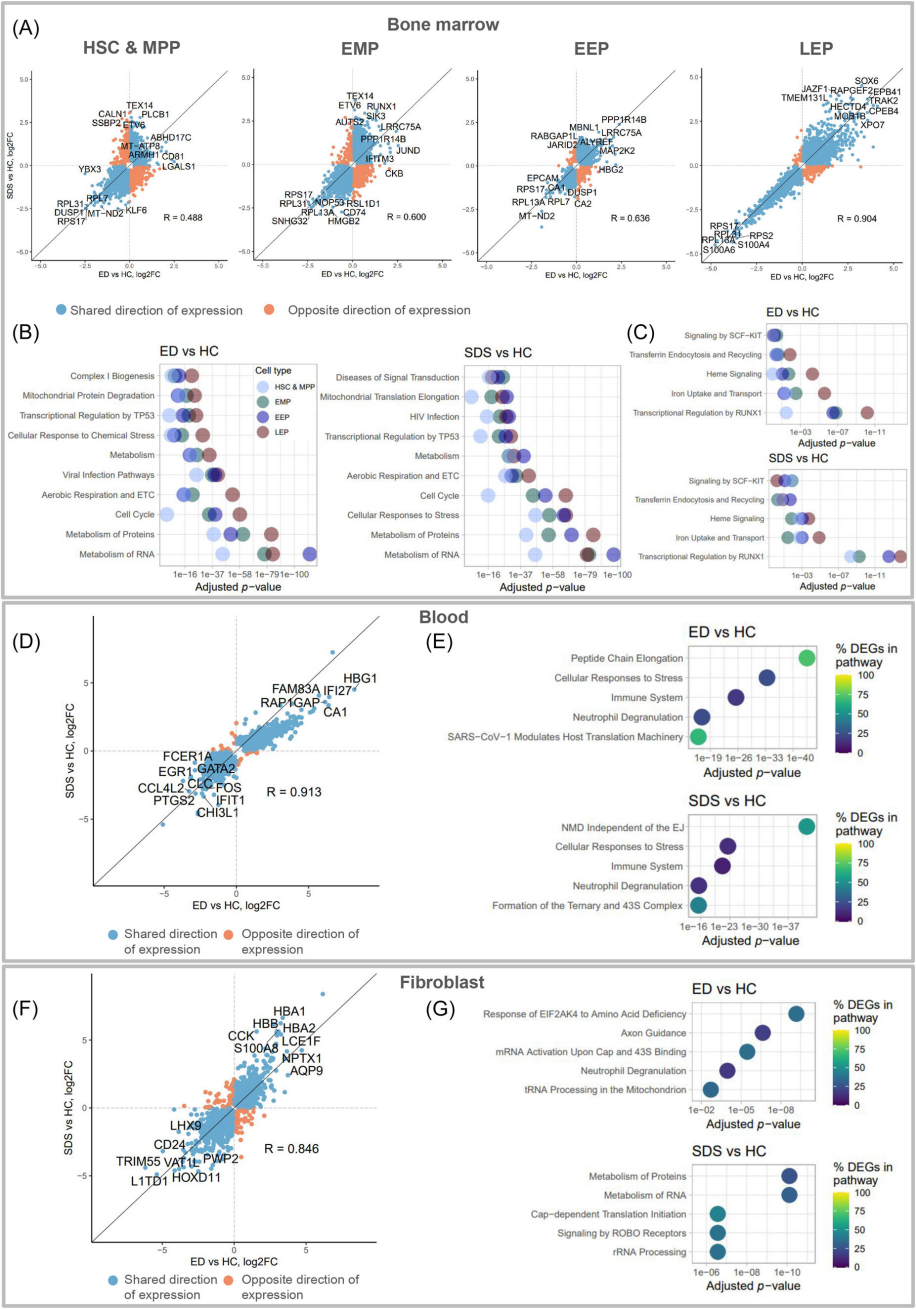
**Transcriptional landscape of bone marrow (BM) erythroid progenitors, peripheral blood cells, and fibroblasts in ERCC6L2 disease (ED) compared to Shwachman–Diamond syndrome (SDS). (A)** Comparison of ED bone marrow failure (BMF) and SDS BMF differentially expressed genes (DEGs) showing log_2_ fold changes (log_2_FC) of expression in ED BMF (*n* samples = 12) and SDS BMF (*n* samples = 4) against healthy controls (*n* samples = 63) in BM hematopoietic stem cell (HSC) and multipotent progenitor cell (MPP) (*n* cells = 370; 30; 27 for healthy control, ED BMF and SDS BMF, respectively), erythroid–myeloid progenitor (EMP) (*n* cells = 1579; 140; 62), early erythroid progenitor (EEP) (*n* cells = 11,826; 777; 232), and late erythroid progenitor (LEP) (*n* cells = 2422; 858; 385). Genes falling close to the diagonal exhibit similar magnitude and direction of differential expression in both diseases, whereas genes deviating from the diagonal reflect differences in the extent of dysregulation between ED and SDS. Blue points indicate genes concordantly regulated in both conditions (upregulated or downregulated relative to controls), while orange points indicate genes regulated in opposite directions between ED and SDS. **(B)** Top 10 non‐redundant pathways across cell types for BM. Enriched pathways were sorted by FDR‐adjusted P‐values P_adj_. Redundant pathways (pathways containing DEGs of which more than half of the DEGs are members of a pathway with a smaller P_adj_) and pathways not enriched for one of the cell types were filtered out. From the remaining pathways, the top 10 based on the smallest P_adj_ across cell types were plotted. **(C)** Hematopoietic‐ and erythroid‐specific pathway enrichment in ED BMF and SDS BMF. Reactome pathway enrichment analysis focusing on pathways related to hematopoiesis and erythropoiesis in bulk blood RNA‐seq data. Pathways were selected based on lineage relevance and the presence of multiple significantly differentially expressed genes, thereby excluding pathways driven by single‐gene effects. Shown are pathways significantly enriched in ED BMF and SDS BMF compared to healthy controls, with adjusted P‐values indicated. **(D)** Comparison of ED BMF and SDS BMF DEGs showing log_2_FC of expression in ED BMF (*n* = 28) and SDS BMF (*n* = 7) against healthy controls (*n* = 11) in blood samples. **(E)** Top five enriched pathways in ED BMF and SDS BMF compared to healthy controls in blood samples. **(F)** Comparison of ED and SDS DEGs showing log_2_FC of expression in ED (*n* = 74) and SDS (*n* = 55) against healthy controls (*n* = 68) in fibroblast samples. **(G)** Top five enriched pathways on ED and SDS compared to healthy controls in fibroblasts. FDR, false discovery rate; HC, healthy control; *R*, Pearson correlation coefficient. DEGs, genes with P_adj_ < 0.05 in the differential expression (DE) analysis results. Enriched pathways, pathways with P_adj_ < 0.05 in pathway analysis results. DEGs were obtained using MAST for BM in **(A)** and using DESeq2 for blood in **(D)** and fibroblast **(F)** and enriched pathways were obtained using enrichR for BM **(B, C)**, blood **(E)**, and fibroblasts **(G)**.

**Figure 3:**
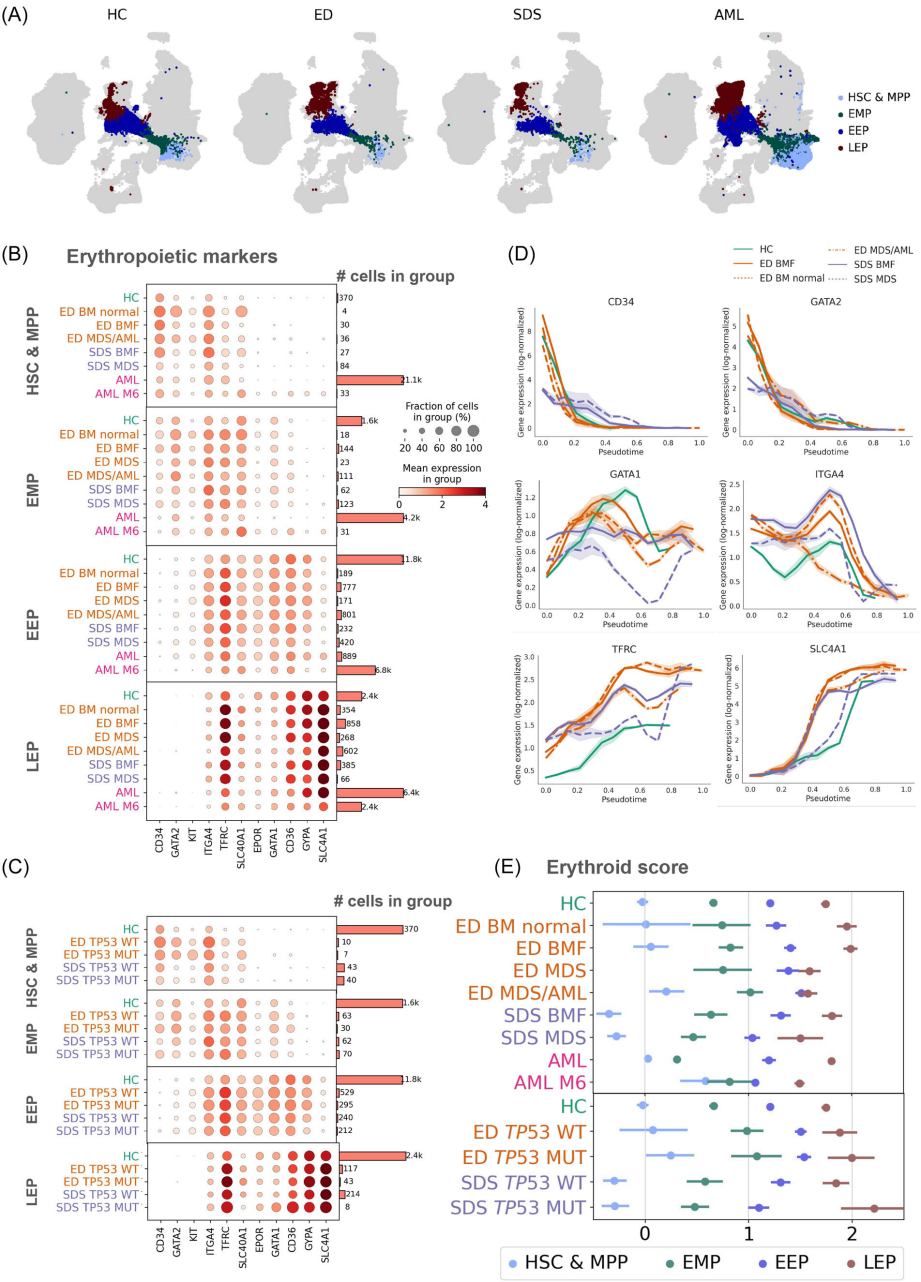
**Erythropoietic cells, erythropoietic markers, and erythropoietic scores for different disease states and *TP53* mutation statuses in ERCC6L2 disease (ED) and Shwachman–Diamond syndrome (SDS) compared to healthy controls. (A)** Erythropoietic cells highlighted (in colors) in the UMAP plot of all cells (in gray). **(B, C)** Expression of erythropoietic markers and number of cells, both across erythropoietic cell types in cells of different **(B)** disease states and **(C)** *TP53* mutation statuses. **(D)** Expression of erythropoietic markers along erythropoietic differentiation identified by pseudotime analyses. Diffusion pseudotime was calculated with CellOracle for each lineage separately. **(E)** Erythroid scores (circles) with 95% CI (lines) calculated by bootstrapping with 10,000 resamples across erythropoietic cell types in cells of different disease states and *TP53* mutation status. Erythroid scores were calculated by Scanpy's implementation of Seurat's cell score calculation using a previously published erythroid‐curated gene list. The score portrays the average expression of an erythroid‐curated set of genes after subtraction by the average expression of a randomly sampled reference set of genes. AML, acute myeloid leukemia; BM, bone marrow; BMF, bone marrow failure; EEP, early erythroid progenitor; EMP, erythroid–myeloid progenitor; HC, healthy control; HSC, hematopoietic stem cell; LEP, late erythroid progenitor; MDS, myelodysplastic syndrome; MPP, multipotent progenitor cell.

For BM samples, we applied single‐cell RNA sequencing (scRNA‐seq, 3′ 10X Genomics) and integrated the data using single‐cell variational inference (scVI).^19^ Cell types were annotated with single‐cell ANnotation using Variational Inference (scANVI)^20^ guided by a hematopoietic marker database^21^ (Supporting Information S2: Supplemental Data, Supporting Information S1: Figure 2). This yielded 32 distinct cell types, including all major erythroid stages: hematopoietic stem cells and multipotent progenitor cells (HSCs and MPPs), erythroid–myeloid progenitors (EMPs), early erythroid progenitors (EEPs), and late erythroid progenitors (LEPs) (Figure 1C). Cell type annotation was examined with known cell type markers, which showed expected expression across cell types (Supporting Information S1: Figure 3A,B) and by comparing the cell type proportions across conditions, which revealed anticipated differences between the conditions, including accumulation of HSCs and MPPs and early progenitor cells in AML compared to the other conditions (Supporting Information S1: Figure 3C–F).

**Figure 4:**
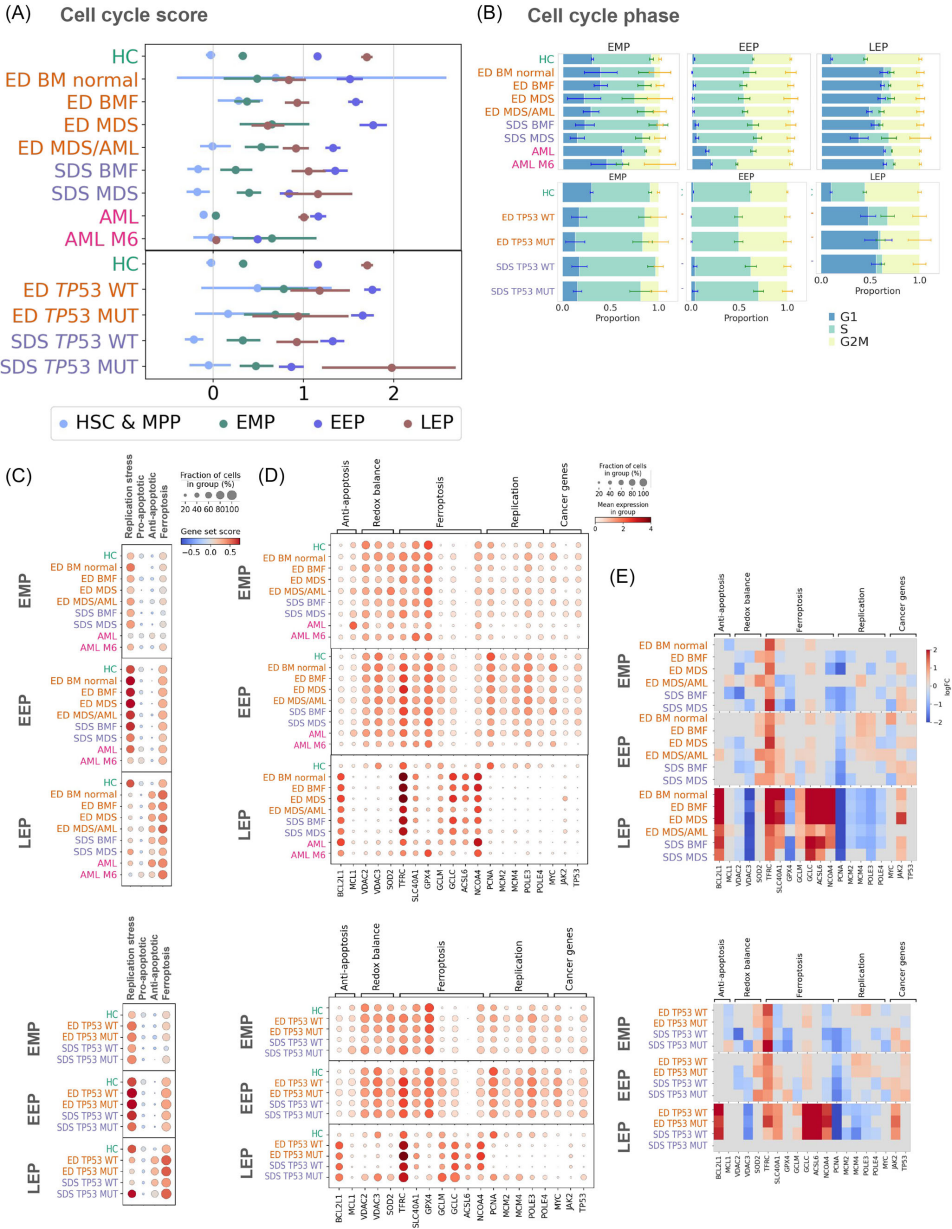
**Cell cycle scores, cell cycle phase proportions, and cell death scores with their differentially expressed key genes. (A)** Cell cycle scores (circles) with 95% CI (lines) calculated by bootstrapping with 10,000 resamples across erythropoietic cell types in cells of different disease states and *TP53* mutation status. Cell cycle scores were calculated by Scanpy's implementation of Seurat's cell score calculation using a previously published cell cycle gene list. The score portrays the average expression of a cell cycle set of genes after subtraction by the average expression of a randomly sampled reference set of genes. **(B)** Cell cycle phases across erythropoietic cell types in cells of different disease states and *TP53* mutation status. Cell cycle phases were calculated using Scanpy's cell cycle scoring, and 95% CI were obtained by bootstrap with 10,000 resamples. Shwachman–Diamond syndrome (SDS) *TP53*‐mutated late erythroid progenitors (LEPs) were too low in number to be included in the analysis. **(C)** Replication stress, pro‐ and antiapoptotic and ferroptotic gene set scores across erythropoietic cell types in cells of different disease states, and *TP53* mutation status. These scores were calculated by Scanpy's implementation of Seurat's cell score calculation using previously published pro‐ and antiapoptotic and ferroptosis gene lists. **(D)** Expression of key genes involved in ferroptosis, apoptosis, and replication across different disease states and *TP53* mutation status. **(E)** Heatmaps showing log_2_FC of differential expression (DE) results of genes presented in **(D)** across different disease states and *TP53* mutation status compared to healthy controls. DE analysis was performed using MAST. AML, acute myeloid leukemia; BM, bone marrow; BMF, bone marrow failure; ED, ERCC6L2 disease; EEP, early erythroid progenitor; EMP, erythroid–myeloid progenitor; HC, healthy control; HSC, hematopoietic stem cell; LEP, late erythroid progenitor; MDS, myelodysplastic syndrome; MPP, multipotent progenitor cell.

To identify the *TP53*‐mutated and wild‐type cells, we applied targeted single‐cell genotyping (scAmp‐seq) within the same ED and SDS samples used for scRNA‐seq, using *TP53*‐specific primers (Supporting Information S3: Table 4) and following established protocols^22^, ^23^ (Supporting Information S2: Supplemental Data, Supporting Information S1: Figure 4). Cells were defined as *TP53* wild‐type only if for each *TP53* variant of the sample the mutation status of the cell was wild‐type, and as *TP53* mutated if the mutation status of at least one *TP53* variant was mutated. Cells with indeterminate *TP53* mutation status were excluded from the analyses. The fraction of identified *TP53*‐mutated cells correlated with the VAF determined in the clinical diagnostics (Pearson correlation *r* = 0.77 and *r* = 0.74 across all cell types and erythropoietic cells, respectively, Supporting Information S1: Figure 4F), in line with results from similar targeted genotyping protocols.^23^ To evaluate the specificity of our pipeline, we applied our pipeline on known wild‐type positions (i.e., positions on *TP53* for which the patients did not carry a mutation), which showed that our pipeline identifies only few false positives at the read level, and almost no false positives at the mRNA transcript and cell levels (specificity > 99.96% on the read, mRNA transcript, and cell level, Supporting Information S1: Figure 4E, Supporting Information S3: Table 5). The *TP53* mutation statuses of cells were mapped to the scRNA‐seq data via shared cell barcodes.

To examine the chromosomal aberrations identified in clinical diagnostics in our scRNA‐seq data, we classified cells as copy number variation (CNV)‐positive, if they showed CNVs in inferCNV^24^ that corresponded to the clinically detected aberrations. All other cells were designated as CNV‐negative. We further performed the following analyses for BM cells: differential expression (DE) with MAST,^25^ pathway analyses using enrichR^26^ with Reactome database,^27^ calculating scores^28^ using erythroid‐curated,^29^ cell cycle,^29^ replication stress,^30^ pro‐ and antiapoptotic^31^ and ferroptosis^32^ gene lists, pseudotime using Genes2Genes^33^ with diffusion pseudotime,^34^ gene regulatory network (GRN) using CellOracle,^35^ supervised gradient boosting model,^36^ and statistical tests.

**Figure 5:**
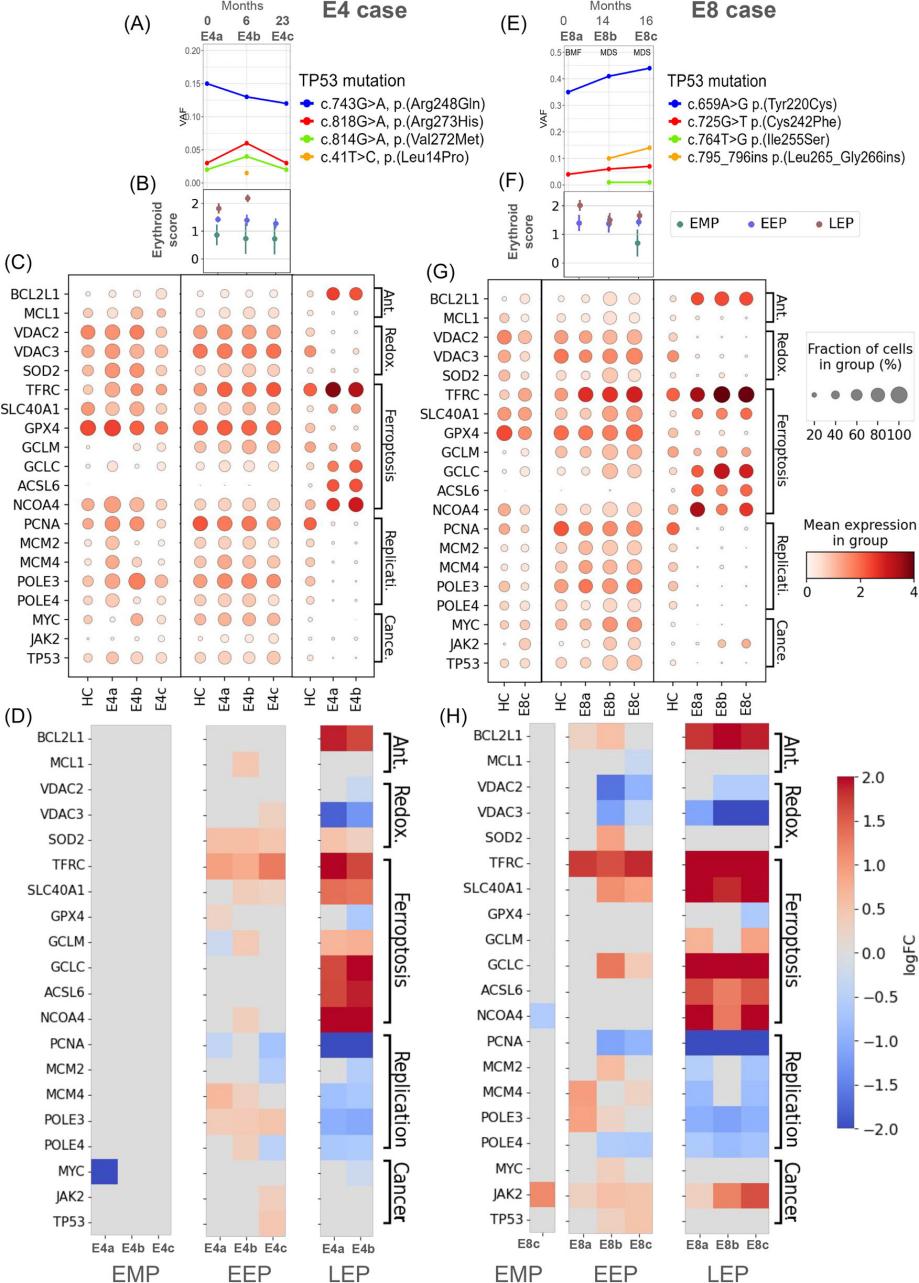
**Longitudinal analysis of erythroid lineage and *TP53* mutation dynamics in ERCC6L2 disease (ED).** Longitudinal analysis of **(A–D)** case E4 and **(E–H)** E8 with three consecutive bone marrow (BM) samples collected. **(A, E)** *TP53* variant allele fractions (VAFs) across timepoints. **(B, F)** The erythroid score across erythroid–myeloid progenitor (EMP), early erythroid progenitor (EEP), and late erythroid progenitor (LEP) stages. Erythroid scores were calculated by Scanpy's implementation of Seurat's cell score calculation using a previously published erythroid‐curated gene list. **(C, G)** Expression of representative ferroptosis‐ and stress‐associated genes. **(D, H)** Heatmaps with log_2_FC of differential expression (DE) results compared to healthy controls. Cell types with less than five cells were removed from the analyses. DE analysis was performed using MAST. HC, healthy control.

For PB cells and fibroblasts, 3′ mRNA sequencing was performed with Illumina NextSeq. 500/550 and aligned to GRCh38.p13^37^ (Supporting Information S2: Supplemental Data). DESeq2^38^ was used for DE (Supporting Information S1: Figure 5), and enrichR^26^ for pathway analysis using Reactome database.^27^ For PB, we also performed deconvolution using CIBERSTORTx.^39^ Across BM, PB, and fibroblast samples, genes were considered differentially expressed if P_adj_ < 0.05 and absolute log‐fold change |log_2_FC| > 0.25. In addition, we predicted the nonsense‐mediated decay (NMD) efficacy^40^, ^41^ for *ERCC6L2*. Full methods are described in the Supporting Information S2: Supplemental Data.

### Data sharing statement

Count tables and processed scRNA‐seq data from this study are deposited at Array Express under accession number E‐MTAB‐16984. The raw data (FASTQ files) are deposited at the Finnish FEGA (Federated European Genome–Phenome Archive) under accession numbers EGAD50000002724 (scAmp‐seq) and EGAD50000002725 (scRNA‐seq), where data access will be evaluated by the Data Access Committee (DAC), and after which the sensitive data can be analyzed in its secure and private cloud workspace in case data access is granted. Python and R scripts used to analyze and plot the data are available at GitHub: https://github.com/lalangohr/ERCC6L2-scRNA/.

## RESULTS

### Truncating *ERCC6L2* mutations escape nonsense‐mediated decay and exhibit expression beyond hematopoietic tissue

**Figure 6:**
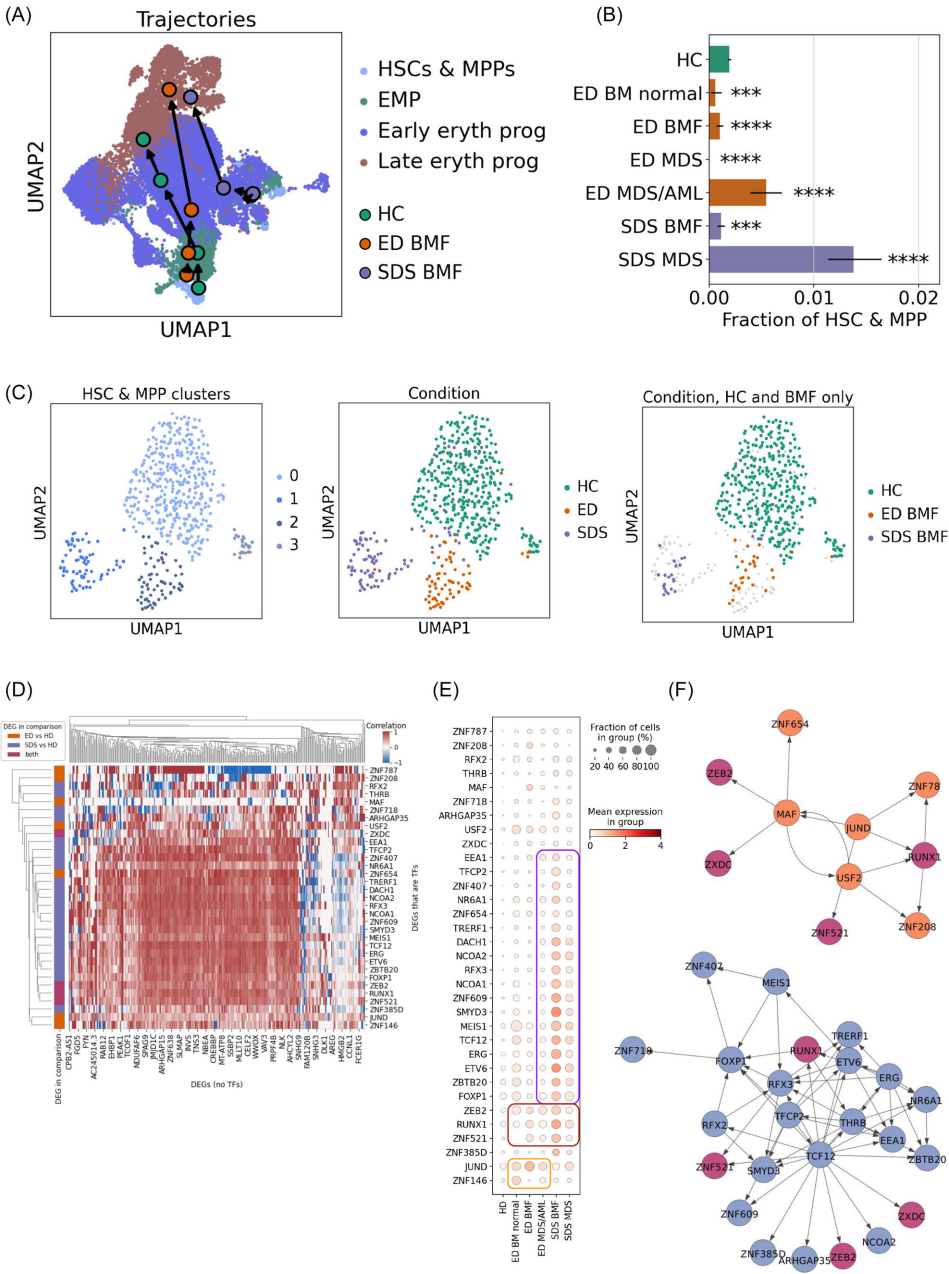
**Differentiation trajectories of erythropoietic cells, proportions of hematopoietic stem cell (HSC) and multipotent progenitor cell (MPP), and transcription factor analyses in HSC and MPP. (A)** Differentiation trajectories of erythropoietic cells in ERCC6L2 disease (ED) bone marrow failure (BMF), Shwachman–Diamond syndrome (SDS) BMF, and healthy controls depicted on a UMAP plot of these cells, after regressing out the *TP53* mutation status and cell cycle scores as well as patient/donor sex and age from gene expression levels. Differentiation trajectories were inferred using PAGA. **(B)** Proportion of HSC and MPP in each sample group. P‐values and 95% CI were obtained by performing bootstrap with 10,000 resamples. ^∗∗∗^P < 0.001; ^∗∗∗∗^P < 0.0001. **(C)** HSC and MPP clusters obtained after regressing out factors listed in (A), then clustering HSC and MPP, visualized as a UMAP plot. Cells colored by clusters (left), condition (middle), and highlighting cells in ED BMF, SDS BMF, and healthy controls (right). **(D)** Heatmap showing Pearson correlation of differentially expressed transcription factors (TFs) and non‐TF differentially expressed genes (DEGs). DEGs were obtained comparing HSC and MPP in ED BMF and SDS BMF to healthy controls. DE analysis was performed using MAST. **(E)** Expression of differentially expressed TFs in HSC and MPP in ED BMF and SDS BMF compared to healthy controls. **(F)** Interactions of differentially expressed TFs in gene regulatory networks of HSC and MPP in ED BMF and SDS BMF. Gene regulatory networks were built using CellOracle. In **(D–F)**, orange indicates TFs uniquely altered in ED, purple in SDS, and red indicates shared between both diseases. EMP, erythroid–myeloid progenitor; HC, healthy control; MDS, myelodysplastic syndrome.

*ERCC6L2* p.Ile475ThrfsTer36 causes a premature termination codon (PTC) 105 nucleotides downstream of the mutation, with 3122 nucleotides between the premature and normal stop codon. Classically, RNAs containing truncating mutations are targeted for degradation by NMD, but they may also escape NMD or be targeted with reduced NMD efficiency.^40^, ^41^ We examined the expression of *ERCC6L2* in the BM and PB cells (affected tissues) and skin fibroblasts (clinically unaffected tissue^5^). Despite the presence of biallelic truncating mutations in *ERCC6L2*, transcripts remained detectable in ED samples (Supporting Information S1: Figure 6A,B). We observed higher *ERCC6L2* expression in the compound heterozygous than in homozygous carriers in fibroblasts (Supporting Information S1: Figure 6A). In SDS, *SBDS* underexpression was visible in fibroblasts and BM compared to controls (Supporting Information S1: Figure 6A).

To reconcile the discrepancy of observing *ERCC6L2* expression despite the PTC being located 3122 nucleotides upstream of the stop codon suggesting highly efficient NMD, we quantified the expected NMD efficacy computationally.^40^, ^41^ Predicted NMD efficacy at *ERCC6L2* PTC was >0.61,^41^ indicating strong (score > 0.52), but not full (score 1) mRNA degradation. Taken together, the germline mutations in both *ERCC6L2* and *SBDS* genes lower the amount of their mRNA transcripts, compatible with partial NMD or altered mRNA surveillance.

### Somatic *TP53* mutations in ED and SDS arise in HSCs or early progenitor cells

**Figure 7:**
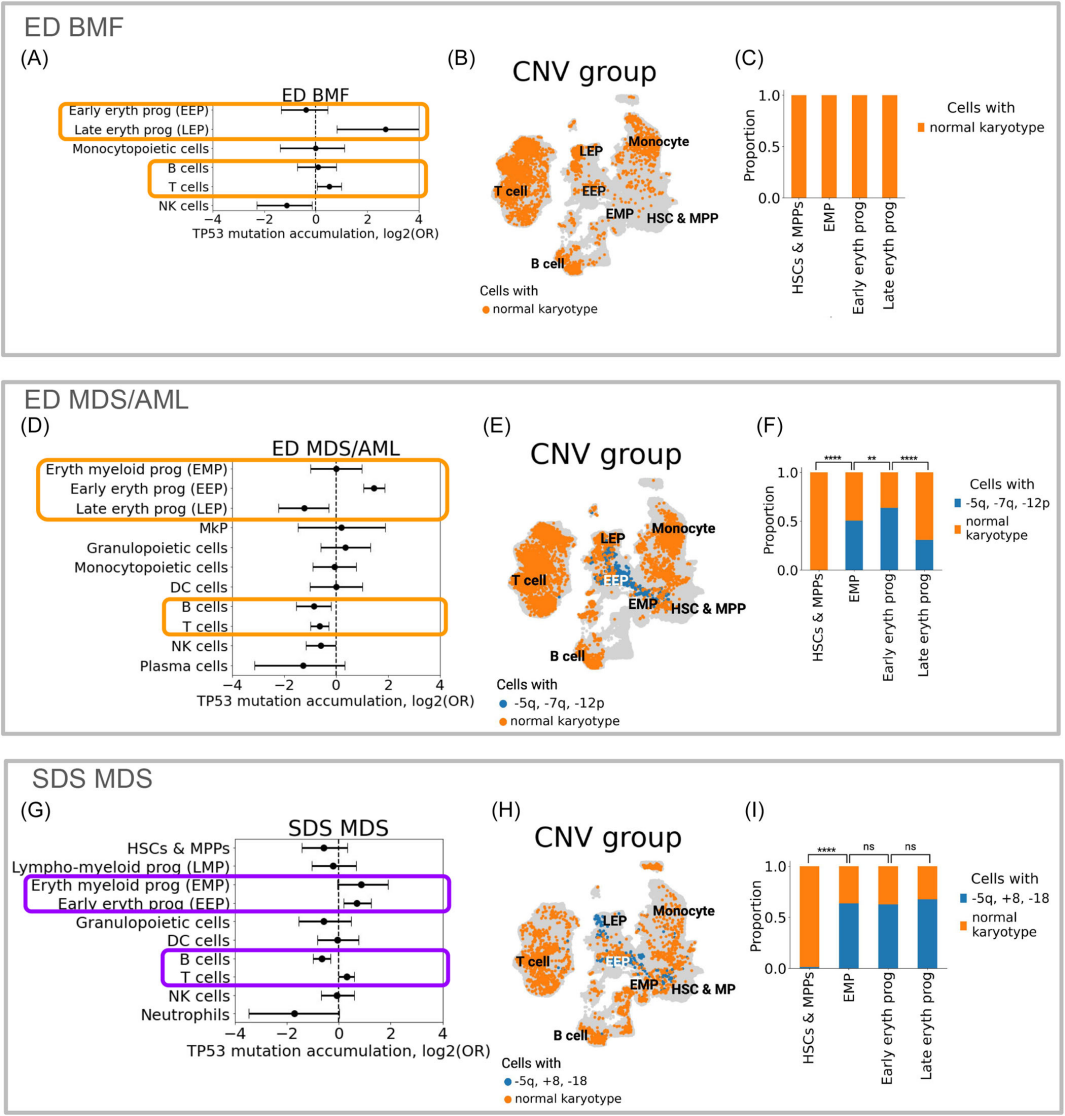
**Accumulation of *TP53* mutations and chromosomal changes in two patient samples with complex karyotype: ERCC6L2 disease (ED) bone marrow failure (BMF) (E2–E8a), ED myelodysplastic syndrome/acute myeloid leukemia (MDS/AML) (E10), and Shwachman–Diamond syndrome (SDS) MDS (SDS5b). (A)** Accumulation and reduction of *TP53*‐mutated cells in different cell types in ED BMF (*n* = 8 samples from six patients), depicted by positive and negative log_2_‐transformed odds ratio (OR) values, log_2_(OR), respectively, reflect relative enrichment or reduction rather than absolute cell numbers. **(B)** Cells without CNVs in ED BMF (E4b) highlighted (in orange) on a UMAP of all cells (in gray). **(C)** Proportion of cells with and without CNVs per cell type in ED BMF (E4b). **(D)** Accumulation and reduction of *TP53*‐mutated cells in different cell types in ED MDS/AML (*n* = 1 sample from one patient), depicted by positive and negative log_2_(OR) values, respectively. **(E)** Cells with and without CNVs in ED MDS/AML (E10) highlighted (in blue and orange, respectively) on a UMAP of all cells (in gray). **(F)** Proportion of cells with and without CNVs per cell type in ED MDS/AML (E10). **(G)** Accumulation and reduction of *TP53*‐mutated cells in different cell types in SDS MDS (*n* = 2 samples from one patient), depicted by positive and negative log_2_(OR) values, respectively. **(H)** Cells with and without CNVs in SDS MDS (SDS5b) highlighted (in blue and orange, respectively) on a UMAP of all cells (in gray). **(I)** Proportion of cells with and without CNVs per cell type in SDS MDS (SDS5b). Accumulation and reduction of *TP53*‐mutated cells in different cell types in **(A)**, **(D)**, and **(G)** were calculated by Fisher's exact *t*‐tests (two‐sided). Only cell types with at least five cells per cell type and *TP53* mutation status are shown. CNVs depicted in **(B)**, **(E)**, and **(H)** were identified using inferCNV. Significances in **(F)** and **(I)** were calculated by a proportion test. ^∗∗^P_adj_ < 0.01; ^∗∗∗∗^P_adj_ < 0.0001; ns, non‐significant P_adj_ > 0.05. CNV, copy number variation; EEP, early erythroid progenitor; EMP, erythroid–myeloid progenitor; HSC, hematopoietic stem cell; LEP, late erythroid progenitor; MDS, myelodysplastic syndrome; MPP, multipotent progenitor cell.

**Figure 8:**
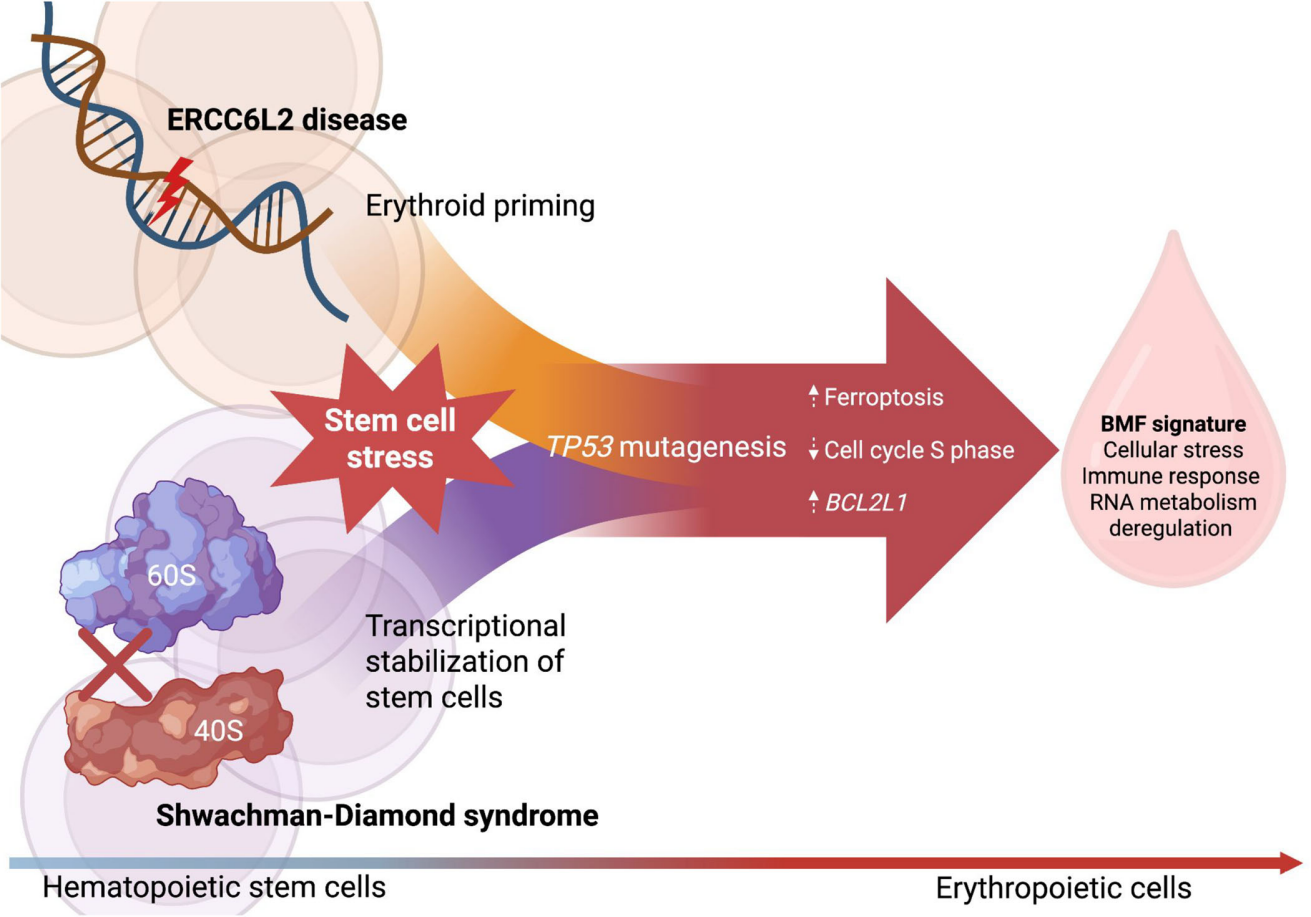
**ERCC6L2 disease (ED) and Shwachman–Diamond syndrome (SDS) show distinct stem cell identity converging into shared erythroid stress.** Overall, our results show that while ED and SDS diverge in early hematopoietic disruption, they converge into a shared bone marrow failure (BMF) phenotype driven by erythroid stress that consists of ferroptosis, cell cycle S‐phase reduction, and prominent *BCL2L1* overexpression. Image created with BioRender.

To study the transcriptomic changes while distinguishing between *TP53*‐mutated and wild‐type cells, we integrated BM scRNA‐seq data from ED, SDS, and AML patients, as well as controls and determined *TP53* mutation status for ED and SDS cells (Methods). The resulting dataset consists of 385,100 cells spanning 32 distinct cell types representing all hematopoietic cell lineages (Figure 1C) consistent with recent single‐cell studies on BM.^42^, ^43^ ED and SDS in the BMF phase closely resembled controls in cell type proportions (Supporting Information S1: Figure 3), in line with clinical observations before malignant transformation.^5^, ^44^ We genotyped the *TP53* mutation status of 1.5%–61.4% ED and SDS cells per sample and mutation site, identifying 26,655 *TP53* wild‐type and 4239 *TP53*‐mutated cells (Figure 1C, Supporting Information S2: Supplemental Methods, Supporting Information S1: Figure 3B). Consistent with bulk sequencing findings in SDS showing widespread *TP53*‐mutated clones,^45^ our study provides the first single‐cell evidence of this phenomenon in ED and maps *TP53*‐mutated cells across erythroid, myeloid, and lymphoid compartments (Figure 1D), indicating that *TP53* mutations arise in HSCs or early progenitor cells (Supporting Information S1: Figures 7 and 8).

### Distinct germline defects lead to shared stress phenotypes in ED and SDS

Since ED develops into erythroid leukemia specifically,^4^, ^5^ we focused on the BM erythroid lineage. When comparing differentially expressed genes (DEGs) between the two diseases, the most pronounced transcriptional differences between ED and SDS emerged in early hematopoietic stages with the disparity progressively diminishing during erythropoiesis (Pearson correlation *r* = 0.5, *r* = 0.6, *r* = 0.6, and *r* = 0.9 for HSC and MPP, EMP, EEP, and LEP, respectively, Figure 2A, Supporting Information S3: Tables 6–9). Pathway analyses along erythropoiesis revealed shared stress signatures (Figure 2B, Supporting Information S3: Tables 10–13). We then refined the analysis to hematopoietic‐ and erythroid‐related pathways, which underscored shared dysregulation of fundamental lineage programs, including growth factor signaling, iron handling, heme metabolism, and transcriptional control, across stem and erythroid progenitor stages (Figure 2C). Concordantly, transcriptomic analyses of PB cells uncovered striking similarities between ED and SDS (Figure 2D, Supporting Information S3: Table 14), and showed upregulation of genes involved in cellular stress responses and immune pathways (Figure 2E, Supporting Information S3: Table 15), consistent with prior studies on inherited BMF syndromes.^46^ Deconvolution of PB bulk RNA‐seq showed known differences in the blood cell compositions such as dominant neutropenia in SDS (P_adj_ = 0.013, two‐sided Wilcoxon rank‐sum) and varying, modest alterations in ED (Supporting Information S1: Figure 5C,D). Patient‐derived fibroblasts, lacking overt disease phenotypes or *TP53* mutations, exhibited transcriptional divergence between ED and SDS (Figure 2F, Supporting Information S3: Table 16). ED fibroblasts showed pathway enrichment related to amino acid sensing via the GCN2 response and axon guidance (Figure 2G, Supporting Information S3: Table 17), possibly reflecting generalized adaptation to chronic metabolic or proteostatic stress. SDS fibroblasts demonstrated broader alterations in RNA and protein metabolism (Figure 2G, Supporting Information S3: Table 17). These findings show that ED and SDS share stress‐related transcriptional features in LEPs and PB cells, while fibroblasts and early hematopoietic progenitors reveal germline‐specific differences independent of disease or *TP53* mutation.

### Aberrant erythroid priming distinguishes ED from SDS during early differentiation

To examine the transcriptional changes in ED and SDS in more detail, we first assessed the expression of erythropoiesis markers along erythropoietic differentiation (Figure 3A–C, Supporting Information S2: Supplemental Methods). *CD34*, *GATA2*, and *GATA1* followed their expected expression pattern in both diseases (Figure 3B–D), whereas *TFRC* showed the most aberrant expression in ED BMF compared to controls (Figure 3B–D, expression dissimilarity *d* = 1.03, 95% CI [0.97, 1.08], Supporting Information S3: Table 18), which showed aberrant expression also in SDS BMF (*d* = 0.83, 95% CI [0.72, 0.93] for *TFRC*, Supporting Information S1: Figure 9, Supporting Information S3: Table 18).

Interestingly, we observed upregulation of erythroid‐associated transcription measured by erythroid scores^29^ in ED but not in SDS compared to controls (Figure 3E, Supporting Information S1: Figure 11). Unlike the expression of individual erythroid marker genes, which indicate lineage identity, the erythroid score reflects global transcriptional maturation along the erythropoietic trajectory.^29^ ED cells showed a higher erythroid score than SDS, suggesting an accentuated commitment toward erythroid differentiation. Among EEPs, the score for ED with normal BM cellularity was lower than for all other ED disease stages and the erythroid score for the MDS/AML case was higher than in all other conditions and diagnoses even exceeding that of the AML M6 case (P < 0.05, P < 0.01, and P < 0.0001, respectively, bootstrap test with 10,000 resamples, Figure 3E, Supporting Information S3: Table 19). The elevated erythroid scores in EEPs in all ED disease stages, except in the ED case with normal BM, normal cellularity, and normal blood counts, might reflect a compensatory upregulation of erythroid transcription under stress. Divergent erythroid scores in ED and SDS point to qualitative differences in differentiation, with ED cells displaying a stronger erythroid transcriptional commitment than SDS or controls.

### ED and SDS LEPs converge on shared cell cycle and ferroptotic stress signatures that persist despite *TP53* mutation

To further investigate erythroid differentiation in ED and SDS, we analyzed cell cycle dynamics and stress‐related gene expression in erythroid progenitor populations. Cell cycle scores^29^ were higher for all ED disease stages compared to control in EEPs (P < 0.0001, bootstrap test with 10,000 resamples, Figure 4A, Supporting Information S1: Figure 10, Supporting Information S3: Table 20), indicating upregulation of cell cycle–associated genes. The distribution of cells across cell cycle phases was similar between *TP53*‐mutated and wild‐type cells (Figure 4B). In contrast, we detected a marked reduction in cell cycle scores in LEPs for all ED disease stages compared to control (P < 0.0001, Figure 4A), consistent with downregulation of cell cycle transcription.

DE analyses further supported these findings, uncovering enrichment of cell cycle ‐related genes in all erythropoietic cell types in both diseases (Figure 2B, Supporting Information S3: Tables 6–9). Examining cell cycle phases revealed that the proportion of S‐phase cells approached controls in EMPs and EEPs (45%–76% in ED and SDS, 61% in controls; and 50%–61% in ED and SDS, 59% in controls, respectively, Figure 4B, Supporting Information S3: Table 21). However, S‐phase cells were drastically reduced in LEPs, which resided mostly in G1 (6%–30% in ED and SDS, 34% in controls; 38%–63% in ED and SDS, 10% in controls, respectively, Figure 4B). Notably, the accumulation of cells in G1 with reduced S‐phase representation mirrors cell cycle restriction observed in *Ercc6l2*‐deficient murine cells, where replication stress constrains cell cycle progression in hematopoietic progenitors.^15^ These results suggest a change in cell cycle dynamics and erythroid differentiation in both ED and SDS, especially in LEPs with a decrease of S phase and an increase of G1 phase, a pattern reminiscent of erythroid differentiation block seen in erythroid leukemia.^47^

Given the aberrant cell cycle dynamics in LEPs and the consistently elevated expression of *TFRC*, encoding CD71, a key marker of iron uptake and ferroptosis sensitivity, we next investigated whether other iron metabolism genes were also affected (Figure 4C,D). DE analyses confirmed overexpression of *TFRC* in patient cells in both diseases compared to controls throughout erythropoietic cell types (Figure 4D,E, Supporting Information S3: Tables 6–9). Interestingly, accumulation of CD71^+^ immature erythroid progenitors has also been reported in Ercc6l2‐deficient murine and human hematopoietic models, consistent with impaired erythroid maturation and increased iron demand at early erythroid stages.^15^ The overexpression of *TFRC* in our patient cells was accompanied by downregulation of *GPX4*, a central suppressor of ferroptosis^48^ in both ED and SDS LEPs (Figure 4D,E). In addition, *VDAC2* and *VDAC3*, which regulate mitochondrial ROS and iron flux,^49^, ^50^ were downregulated in LEPs (Figure 4D,E). Concomitant with upregulated ferroptosis, antiapoptotic genes were upregulated in both ED and SDS LEPs (Figure 4C–E, Supporting Information S1: Figure 11). Especially antiapoptotic *BCL2L1* (encoding for BCL‐XL), which is downstream of *EPO* and is vital for erythropoiesis,^51^ was overexpressed in ED and SDS LEPs (Figure 4D,E). These observations suggest iron‐redox imbalances and increased reliance on *BCL2L1* in both ED and SDS. While acute Ercc6l2 loss in murine cells has been shown to trigger p53‐dependent apoptosis and cell cycle arrest in stem and progenitor cells,^15^ our analyses of patient‐derived erythroid cells did not reveal a dominant apoptotic signature. Instead, stress responses in ED and SDS appeared to shift toward ferroptosis susceptibility and reliance on antiapoptotic programs, particularly at the LEP stage.

Since ERCC6L2 is implicated in replication^9^ and cell cycle phase perturbances were evident in LEPs, we next evaluated replication‐associated gene expression across erythropoiesis. In EEPs, *MCM* and *POLE* genes that participate in replication were overexpressed in both diseases, but their expression declined in LEPs (Figure 4D,E), potentially reflecting replication stress or failure to resolve cell cycle progression, a known driver of DNA damage and ferroptosis sensitivity.^49^, ^50^ *PCNA* was downregulated in both EEPs and LEPs in ED and SDS (Figure 4D). Its DE reinforces the presence of replication‐associated stress, as PCNA interacts with ERCC6L2 to support replication fork progression.^9^ This is consistent in ED, where *ERCC6L2* is mutated, but its downregulation in SDS is nonetheless noteworthy. In accordance with the gene‐level changes, replication stress scores were elevated in ED EEPs (P < 0.001, for each ED BM normal, ED BMF, ED MDS, and ED MDS/AML, each compared to HC, bootstrap test with 10,000 resamples, Figure 4C, Supporting Information S3: Table 22) independent of *TP53* mutation status, consistent with loss of Ercc6l2 in murine models.^15^ Replication stress scores declined sharply upon transition to LEPs in ED (Figure 4C). Moreover, we noted increased *JAK2* expression (Figure 4D,E) in LEPs, possibly reflecting adaptive changes in signaling during stressed erythropoiesis.^52^, ^53^

While p53 has been shown to regulate ferroptosis and *GPX4* expression in transformed cancer and leukemia models,^54^ the impact of *TP53* mutations in the context of inherited BMFs remains less defined. Notably, the presence of *TP53* mutations in ED and SDS cells did not restore normal expression of key ferroptosis or cell cycle genes (Figure 4E, Supporting Information S3: Tables 23–26). Thus, *TP53* mutations in these contexts may be a secondary adaptation, insufficient to override the fundamental replication and redox dysregulation imposed by the primary germline *ERCC6L2* and *SBDS* deficiency. Since the *TP53* mutations per se seem not to change the stress signatures in ED cells, we investigated whether we could find markers that indicate disease progression within ED BMF. We applied a supervised gradient boosting model^36^ comparing stable ED BMF cases to ED BMF cases with signs of disease acceleration (increasing erythropoiesis in BM biopsy, *TP53* VAF, or number of clones,^5^ Supporting Information S3: Table 2) but identified no consistent transcriptional markers of disease acceleration in ED PB or BM erythropoietic cells (Supporting Information S1: Figure 12). Together, these findings suggest that LEPs in ED and SDS may exist in a replication‐stressed state with insufficient resolution of cell cycle progression, necessitating antiapoptotic support and predisposing cells to ferroptosis, maintained even in the presence of *TP53* mutations.

### Longitudinal sampling shows ferroptotic stress and its persistence across stable and progressive ED disease courses

To assess the temporal dynamics of erythropoiesis and *TP53* mutation acquisition in ED, we analyzed two cases with longitudinal BM sampling (Figure 5). In case E4, three BM samples spanning 23 months were obtained during clinically stable BMF (Figure 5A). All samples showed modest *TP53* VAFs (≤15%), with a minor additional *TP53* variant (c.41T>C) detected transiently at the second timepoint (Figure 5A). Throughout the follow‐up time, the erythroid expression patterns remained largely unchanged (Figure 5B,C, Supporting Information S3: Table 27). Comparison to healthy controls confirmed this stability: EMPs and EEPs closely resembled healthy controls, whereas LEPs consistently showed elevated ferroptosis marker gene expression and downregulation of replication, together with upregulated *BCL2L1* (Figure 5C,D, Supporting Information S3: Table 27). LEP analysis was limited in the final sample due to low cell yield. Overall, the E4 case represents a stable transcriptional and clonal state in BMF, with no evidence of erythroid trajectory shift despite detectable *TP53*‐mutated clones.

In contrast, case E8 demonstrated disease progression from BMF to MDS over 14 months, coinciding with expansion of the dominant *TP53* clone and acquisition of two additional *TP53* mutations (Figure 5E). A third sample, collected two months after the MDS diagnosis, confirmed clonal persistence. Notably, LEP erythroid scores decreased with the transition from BMF to MDS (P < 0.001, bootstrap test with 10,000 resamples, Figure 5F, Supporting Information S3: Table 28), indicating a shift in dynamics not observed in the stable E4 trajectory. In the E8 case, erythroid trajectory was not transcriptionally static: compared with the BMF sample, EEPs in both MDS samples displayed stronger redox gene downregulation and higher expression of *JAK2*, while ferroptosis‐associated expression patterns were maintained in LEPs across all timepoints (Figure 5G,H, Supporting Information S3: Table 27). EMP evaluation was limited at early timepoints due to cell scarcity.

Together, these longitudinal cases illustrate two trajectories: one with stable hematopoiesis and low *TP53* burden (E4), in which ferroptotic stress remains steady over time, and another with progressive clonal expansion (E8), where stress features become more apparent alongside clinical progression. This aligns with our broader observation that ferroptosis is present early in ED and persists across disease stages.

### Intrinsic differences in stem cell regulation separate ED and SDS in early hematopoiesis

To isolate fundamental changes in erythropoiesis from effects due to *TP53* mutations, cell cycle variation, sex, and age, we regressed out these variables. Following this correction, HSCs, MPPs, and EMPs clustered more closely for ED and controls than for SDS (Figure 6A). Differentiation trajectories showed ED and SDS converging in EEPs, and ultimately aligning closely with controls in LEPs (Figure 6A), mirroring the expression patterns described earlier (Figure 2A) and underscoring intrinsic transcriptional differences in early hematopoiesis. Stem cells are scarce in healthy BM,^55^ and we detected on average six HSCs and MPPs per sample for controls (Figure 6B) and even less in ED and SDS BMF (proportion of HSCs and MPPs 0.10%, 95% CI [0.07%, 0.13%], P < 0.0001 and 0.11%, 95% CI [0.08%, 0.15%], P = 0.0003, respectively, compared to 0.19% in controls; Figure 6B), consistent with HSC exhaustion that leads to BMF. No HSCs and MPPs were detected in ED MDS, reflecting the characteristic hypocellular state.

Focused analysis of HSCs and MPPs revealed distinct clusters for ED, SDS, and controls (Figure 6C), leading us to examine whether transcription factor (TF) dysregulation underlies these early hematopoietic differences. We found partial clustering by disease, but also overlapping transcriptional changes (Figure 6D,E). SDS displayed a broader range of TF dysregulation, with a greater number of DEGs compared to ED (Figure 6D,E). To further assess the relationships between these TFs, we constructed GRNs that highlighted the differentially expressed TFs *JUND, RUNX1, USF2*, and *MAF* in ED (Figure 6F), which may point to coordinated roles in maintaining HSC self‐renewal and potentially regulating early lineage priming.^56^, ^57^, ^58^, ^59^, ^60^ Notably, *RUNX1* and *JUND* interface with *TP53* and oxidative stress pathways,^61^, ^62^, ^63^ potentially contributing to erythroid priming and antiapoptotic signaling observed in ED. In contrast, SDS cells exhibited overexpression of TFs such as *TCF12*, *ETV6*, *ERG*, and *RUNX1* (Figure 6F). These factors likely maintain HSC identity^64^, ^65^, ^66^ and could buffer against premature differentiation, rather than promote lineage skewing. Murine models of Ercc6l2 loss show suppression of *Runx1* that is reversed by concomitant Trp53 loss,^15^ potentially reflecting differences between acute stress responses and adapted transcriptional states observed in patient‐derived cells. Together, these findings support that ED and SDS, despite their shared phenotype of BMF, are rooted in distinct disruptions of stem and progenitor cell identity.

### *TP53*‐mutated cells show erythroid differentiation block in EEPs in ED MDS/AML patient

Finally, to understand how *TP53* mutations and chromosomal alterations contribute to leukemic transformation in ED and SDS, we examined their distribution and impact across differentiation stages. In ED BMF, *TP53* mutations accumulated only in LEPs (odds ratio [OR] = 6.5, 95% CI [1.8, 20.1], P_adj_ = 0.04, P_adj_ > 0.1 for all other cell types, Figure 7A, Supporting Information S3: Table 29). No CNVs were found in the BMF sample with the highest detected number of *TP53*‐mutated cells (E4b, Figure 6B,C, Supporting Information S1: Figure 7), aligning with clinical data (Supporting Information S3: Table 2). This is consistent with models of stepwise clonal evolution in *TP53*‐mutated myeloid disease, in which *TP53* mutations arise before acquisition of chromosomal aberrations and biallelic inactivation drives transformation.^67^

In the ED MDS/AML case (E10), *TP53*‐mutated cells were enriched in EEPs (OR = 2.8, 95% CI [2.1, 3.7], P_adj_ = 6.18 × 10^−13^), while their frequency was significantly reduced in LEPs (OR = 0.4, 95% CI [0.2, 0.8], P_adj_ = 0.03; Figure 7D, Supporting Information S3: Table 29), aligning with clinical reports of erythroid‐predominant AML in ED.^4^, ^5^ This suggests that transformation may involve a differentiation block specifically at the EEP‐to‐LEP transition, rather than a failure of early erythroid commitment. Although p53 normally restrains erythroid progenitor proliferation, the OR‐based enrichment of *TP53*‐mutated cells in EEPs may reflect preferential retention rather than proliferative expansion, occurring in the context of replication stress and impaired downstream progression.^68^ In this patient we also noticed a reduction of *TP53*‐mutated cells in B and T cells (OR = 0.6, 95% CI [0.3, 0.9], P_adj_ = 0.03 and OR = 0.6, 95% CI [0.5, 0.8], P_adj_ = 0.003, respectively, Figure 7D, Supporting Information S3: Table 29) as well as a pre‐B and pro‐B cell depletion (Supporting Information S1: Figure 13), which has been observed in AML.^69^ In cell cycle phases, LEPs in G1 phase were reduced in the ED MDS/AML case compared to the other stages of ED (48% and 61%–63%, respectively, Figure 4B). CNV analyses located losses in chromosomes 5 and 7 in erythroid progenitors (Figure 7E, Supporting Information S1: Figure 14) with increased accumulation in EEPs (proportion test's *z* = 12.15, P = 5.59 × 10^−34^, false discovery rate [FDR] = 3.36 × 10^−33^ in EEPs compared to LEPs Figure 7F, Supporting Information S3: Table 30). Cells with CNVs were more often *TP53*‐mutated than those without CNVs (*z* = 26.4, P = 4.40 × 10^−153^). Together, these findings suggest that in the ED MDS/AML case, *TP53* mutations and chromosomal aberrations stall erythroid differentiation at the EEP stage, potentially driving malignant progression by expanding a population of progenitors with genomic instability. While allelic configuration (mono‐ vs. biallelic *TP53* alteration) could not be determined in this dataset, co‐occurrence of *TP53* mutations with chromosomal losses is compatible with models of biallelic inactivation that drive aggressive myeloid transformation, as previously demonstrated in large clinical cohorts.^67^

In the SDS MDS patient case (SDS5b), we observed enrichment of *TP53*‐mutated cells across the erythroid lineage, though only the enrichment in EEPs reached statistical significance (OR = 1.6, 95% CI [1.1, 2.4], P_adj_ = 0.04, Figure 7G, Supporting Information S3: Table 29). Losses of chromosomes 5 and 18 and gains of chromosome 8 (reported in the clinical patient work‐up; Supporting Information S3: Table 2) were detected throughout the erythroid lineage (Figure 7H,I, Supporting Information S1: Figure 14), contrary to the ED MDS/AML case, where the prevalence of CNVs declined in LEPs (Figure 7F). The SDS MDS patient cells with these CNVs were more often *TP53*‐mutated than those without CNVs (*z* = 27.0, P = 6.75 × 10^−161^). LEPs in G1 phase were reduced in SDS MDS compared to SDS BMF (38% and 54%, respectively, Figure 4B). Together, in the SDS MDS case, *TP53* mutations and CNVs are observed across the erythroid lineage, but low numbers of *TP53*‐mutated cells detected in LEPs (Figure 3C) limited interpretation of their cell cycle state or potential differentiation block.

These observations on single cases are consistent with the clinical depiction of erythroid‐predominant leukemia in ED and align with murine and human models showing that *TP53* loss preferentially affects erythroid differentiation,^70^, ^71^, ^72^, ^73^, ^74^ possibly due to unique replication or redox stress vulnerabilities in erythroid progenitors.

## DISCUSSION

The hallmark of ED is a highly penetrant progression from BMF to erythroid‐predominant, *TP53*‐mutated myeloid malignancy. Given the limited therapeutic options and poor prognosis of *TP53*‐driven leukemias,^13^, ^75^, ^76^ identifying early disease‐associated vulnerabilities in ED is crucial. Thus, we investigated the transcriptomic consequences of *ERCC6L2* deficiency focusing especially on events prior to malignant transformation with an extensive multi‐tissue dataset for the rare disease.

Our findings suggest that ED cells engage a stronger erythroid transcriptional bias, as indicated by a higher erythroid score^29^ early in differentiation, setting them apart from SDS. Interestingly, the erythroid trajectories in ED and SDS unite over maturation: LEPs exhibited signs of ferroptotic stress, redox imbalance, and a potential dependency on *BCL2L1* in both diseases, suggesting shared vulnerabilities (Figure 8). Recognizing the emerging data on sensitivity to BCL‐XL inhibition of *TP53*‐mutated erythroid leukemia cells^18^, ^77^ calls for further studies, also in the BMF context. Ferroptosis, an iron‐dependent, lipid peroxidation‐driven form of cell death, has been implicated in hematologic disorders including BMF syndromes.^78^, ^79^, ^80^ Recent studies indicate that HSCs are particularly susceptible to ferroptosis due to their unique metabolic characteristics including iron processing.^81^, ^82^ In ribosome‐related BMFs, such as SDS, disruptions in ribosomal function can lead to oxidative stress and iron accumulation, further sensitizing cells to ferroptosis.^78^ In ED, ferroptosis may arise from impaired stress response capacity and redox imbalance in erythroid‐biased progenitors. Hence, targeting therapeutically ferroptotic pathways via iron chelation, lipid peroxidation inhibitors, or modulation of glutathione metabolism is worth investigating to alleviate hematopoietic dysfunction.

Consistent with stress pathway dysregulation, we identified GRNs indicated in DNA damage and oxidative stress, undermining the cellular capacity to buffer against DNA damage and contributing to HSC exhaustion. The DE of *RUNX1* emerged as a shared regulatory node in ED and SDS, potentially integrating stress signals and modulating downstream programs in both diseases. Despite distinct root causes of these diseases, both germline defects ultimately impose a bottleneck on erythropoiesis, channeling hematopoiesis into a shared, stressed erythroid transcriptome.

In ED, *TP53*‐mutated clones arise in stem and progenitor cells, supporting the notion that *TP53* mutations provide a selective advantage under chronic stress by sustaining BM cellularity, similar to the adaptations seen in SDS.^11^, ^12^ However, this adaptation appears inherently unstable, as biallelic *TP53* inactivation represents a pivotal step toward leukemic transformation. Recent functional studies in murine models have provided a mechanistic framework for the selective pressure, demonstrating that Ercc6l2 loss induces replication stress and impaired hematopoietic fitness that can be alleviated by Trp53 inactivation at the cost of persistent genomic instability and malignant progression.^15^ Our patient‐derived data extend this framework by showing that despite the presence of *TP53* mutations, stress‐associated transcriptional programs remain prominent within the erythroid lineage. While p53 inactivation is often thought to alleviate differentiation blocks by disabling DNA damage checkpoints,^83^, ^84^ our findings do not support a substantial rescue effect at least within the erythroid lineage, where we did not observe differences in stress signatures between *TP53*‐mutated and wild‐type cells in either ED or SDS. Together, these findings suggest that somatic *TP53* mutations may support cellular persistence without fully normalizing the transcriptional consequences of chronic *ERCC6L2* deficiency, highlighting a distinction between functional rescue and restoration of homeostatic hematopoiesis and underscoring the biological complexity of hematopoietic failure. While our focus was on germline‐driven disease, future studies hopefully elucidate whether stress signatures precede *TP53‐*mutated AML or erythroid AML more generally.

As a limitation of our study, we could not distinguish between monoallelic and biallelic *TP53* mutations due to recognized challenges in genotyping genes with low expression.^22^, ^23^ All analyzed BM samples carried the same Finnish founder *ERCC6L2* mutation, and a compound heterozygous case was only available as fibroblast samples. The geographic and genetic homogeneity possibly limits broader generalization, but also provides a genetically well‐defined cohort with consistent clinical features across mutation types as shown previously,^5^ supporting the robustness of the observed transcriptomic patterns. We also acknowledge the limited sample size and uneven representation of disease stages, which reflect the rarity and newly recognized entity of ED. Nevertheless, our cohort represents the most comprehensive collection currently available and offers an important foundation for future functional studies. Biobanking of well‐characterized samples, combined with advanced single‐cell, genomic, and proteomic approaches, will be essential to validate these findings and elucidate the mechanistic links between ERCC6L2 dysfunction, *TP53* mutations, and leukemic transformation.

Our findings reinforce the importance of studying primary patient material. Patient‐derived samples reveal disease‐relevant mechanisms, such as retained *ERCC6L2* transcripts, or the stepwise accumulation of *TP53* mutations, that may not emerge in knockout models. They also allow interrogation of heterogeneous cell states across disease stages. Here, we provide the first patient‐level single‐cell map of ED and SDS as a resource on these diseases and TP53‐driven leukemogenesis. In conclusion, our study indicates that while ED and SDS diverge in early hematopoiesis, they converge into a shared BMF phenotype driven by erythroid stress that consists of ferroptosis, cell cycle S‐phase reduction, and prominent *BCL2L1* overexpression.

## AUTHOR CONTRIBUTIONS

**Laura Langohr**: Conceptualization; data curation; formal analysis; methodology; software; visualization; writing—original draft; writing—review and editing. **Ilse Kaaja**: Conceptualization; data curation; investigation; formal analysis; software; visualization; writing—original draft; writing—review and editing. **Suvi P. M. Douglas**: Conceptualization; data curation; formal analysis; software; visualization; writing—review and editing. **Hanna Nebelung**: Data curation; formal analysis; software; visualization. **Jessica Koski**: Data curation; formal analysis; software; visualization. **Ina Ikonen**: Investigation; data curation; software; formal analysis. **Lotta Katainen**: Investigation; writing—review and editing. **Katri Maljanen**: Data curation; formal analysis; software; visualization. **Marja Hakkarainen**: Resources. **Tuulia Räisänen**: Investigation. **Riitta Niinimäki**: Resources. **Sakari Kakko**: Resources. **Timo Siitonen**: Resources. **Sadiksha Adhikari**: Resources. **Markus Vähä‐Koskela**: Resources. **Caroline A. Heckman**: Resources. **Jenni Lahtela**: Conceptualization; methodology. **Ulla Wartiovaara‐Kautto**: Conceptualization; supervision; funding acquisition; resources; writing—review and editing. **Esa Pitkänen**: Conceptualization; methodology; supervision; funding acquisition; writing—review and editing. **Outi Kilpivaara**: Conceptualization; supervision; funding acquisition; writing—review and editing.

## CONFLICT OF INTEREST STATEMENT

T.S. received expert fees from Amgen, AbbVie, GSK, Otsuka Pharma, Celgene, and Jansen‐Cilag unrelated to this work. C.A.H. received research funding from BMS/Celgene, Kronos Bio, Novartis, Oncopeptides, Orion Pharma, WntResearch, and the Innovative Medicines Initiative 2 project HARMONY, and expert fees from Amgen and Autolus unrelated to this work. U.W.‐K. received expert fees from Amgen and Incyte unrelated to this work.

## ETHICS STATEMENT

The study was conducted in accordance with the Declaration of Helsinki. The study has been approved by Helsinki University Central Hospital ethics review committee (#206/13/03/03/2016, amendment 2023, and HRUHLAB2). All samples from living individuals are derived after written informed consent.

## FUNDING

This work was supported by the Research Council of Finland (#349760, #322675), iCAN Digital Precision Cancer Medicine Flagship, Sigrid Jusélius Foundation, Cancer Foundation Finland, Children′s Cancer Foundation AAMU, and Finnish Special governmental grant for health sciences and research. This project was also supported by an unrestricted educational grant from Incyte Biosciences and grants from Instrumentarium Science Foundation, Biomedicum Helsinki Foundation, Päivikki and Sakari Sohlberg Foundation, Emil Aaltonen Foundation (S.P.M.D.), K. Albin Johansson Foundation (I.K.), Paulo Foundation, Ida Montin Foundation, Finnish Hematology Association, Blood Disease Research Foundation, Orion Research Foundation sr (I.K. and S.P.M.D.), and iCANDOC doctoral education pilot in precision cancer medicine (H.N.). O.K. is a K. Albin Johansson Cancer Research Fellow, Foundation for the Finnish Cancer Institute. Open access publishing facilitated by the University of Helsinki, as part of the Wiley ‐ FinELib agreement.

## Supporting information

Supporting Information.

Supporting Information.

Supporting Information.

## Data Availability

Count tables and processed scRNA‐seq data from this study are deposited at Array Express under accession number E‐MTAB‐16984. The raw data (FASTQ files) are deposited at the Finnish FEGA (Federated European Genome–Phenome Archive) under accession numbers EGAD50000002724 (scAmp‐seq) and EGAD50000002725 (scRNA‐seq), where data access will be evaluated by the Data Access Committee (DAC), and after which the sensitive data can be analyzed in its secure and private cloud workspace in case data access is granted. Python and R scripts used to analyze and plot the data are available at GitHub: https://github.com/lalangohr/ERCC6L2-scRNA/.
